# Gastric Heterotopic Pancreas Presenting as Abdominal Pain with Acute and Chronic Pancreatitis in the Resected Specimen

**DOI:** 10.1155/2019/2021712

**Published:** 2019-03-06

**Authors:** Joseph Wawrzynski, Lauren De Leon, Samir A. Shah, Alyn Adrain, Lisa J. Goldstein, Edward Feller

**Affiliations:** ^1^Warren Alpert Medical School, Brown University, Providence, RI, USA; ^2^Coastal Medical Group, Providence, RI, USA; ^3^Department of Pathology and Laboratory Medicine, Warren Alpert Medical School, Brown University, Providence, RI, USA

## Abstract

Heterotopic pancreas, also known as ectopic pancreas, is pancreatic tissue located outside the pancreatic parenchyma without vascular or ductal communication with the gland. Ectopic pancreas is rarely symptomatic, typically detected incidentally at surgery or autopsy. Eighty-five to 90% are in the upper GI tract, especially the gastric antrum. We report a 54-year-old man with symptomatic gastric heterotopic pancreas presenting as recurrent, initially undiagnosed, abdominal pain. Surgery revealed heterotopic pancreas including excretory ducts, acini, and islet cells. Evidence of acute pancreatitis was present, marked by inflammation and abscess formation. Chronic pancreatitis was diagnosed by fibrosis and dilated ducts containing proteinaceous material. Submucosal location with normal overlying mucosa on endoscopy increases risks of delayed or missed diagnosis. Complications include GI bleeding, acute or chronic pancreatitis, pancreatic necrosis, pseudocyst, gastric outlet obstruction, perforation, and, rarely, pancreatic carcinoma. This rare disorder mimics more common diseases. Low suspicion, nondiagnostic imaging or endoscopy contribute to frequent diagnostic delay.

## 1. Introduction

Ectopic pancreas is rarely symptomatic. The vast majority of lesions are detected incidentally at surgery or autopsy. Barbosa and colleagues reported approximately 1 incidental case detected per 500 upper abdominal operations; the incidence for all autopsies was 1.7% [1, 2]. Other sources cite an overall incidence of 0.2-0.8% in surgical patients and 0.6-14% of autopsy cases [3–6]. Eighty-five to 90% are found in the upper GI tract, especially the gastric antrum and duodenum [4, 5]. Distal small bowel, omental, or mesenteric locations are less common [2]. Involvement of the mediastinum, lungs, liver, gallbladder, spleen, esophagus, fallopian tubes, or Meckel's diverticulum is very rare [1]. Gastric heterotopic pancreas has been reported to be submucosal in about three-quarters of cases and is located in the muscularis propria or subserosa in the remaining cases [4, 7].

We report a rare case of symptomatic gastric heterotopic pancreas presenting as nonspecific, recurrent abdominal pain with a characteristic submucosal gastric mass on endoscopy. This case is exceptional because the ectopic tissue contained evidence of acute and chronic pancreatitis and abscess formation.

## 2. Case Presentation

A 54-year-old man was admitted with 4-5 days of abdominal pain that began in the left upper quadrant and then migrated subumbilically. The pain was sharp, steady, and of moderate severity. He also described nausea and a low-grade fever. Past medical history included kidney stones and a sigmoid colectomy for diverticulitis 15 years previously. He denied a history of excess alcohol use. His only medication was atenolol 50 mg daily.

On admission, physical examination revealed normal sinus rhythm, normal pulse and blood pressure without postural change, normal temperature, moderate tenderness to palpation in the left supraumbilical and subxiphoid areas without peritonitis, organomegaly, or mass lesion. Stool was negative for occult blood.

Lab results: WBC: 10 x 10^3^/*μ*L, Hgb: 16g/dL, Chemistry 7, liver enzymes, and serum amylase and lipase were within normal limits. Upper GI endoscopy was interpreted as a 5 mm sessile gastric antral polyp with normal gastric mucosa on biopsy. Abdominal CT scan showed mesenteric inflammation surrounding the distal body and proximal antrum of the stomach and adjacent low-density thickening of the stomach wall, measuring up to 18 mm in thickness. He was treated with IV fluids and pain medication. His condition improved and he was discharged home without a definitive diagnosis.

**Figure 1:**
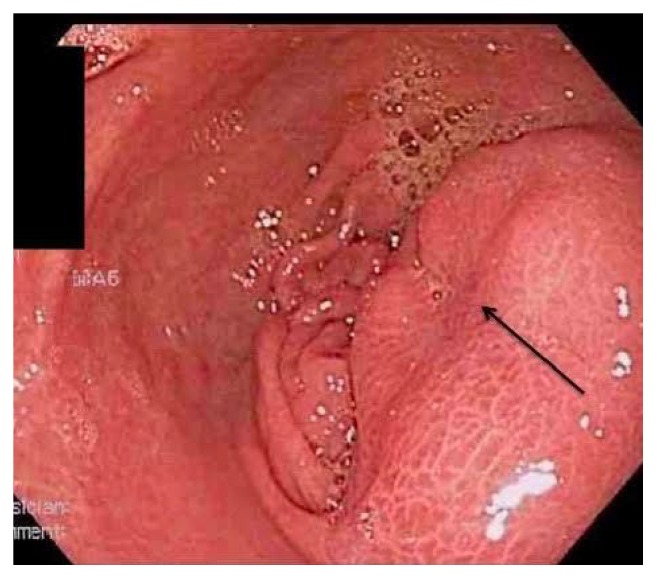
Firm submucosal mass with intact overlying mucosa and a central umbilication.

**Figure 2:**
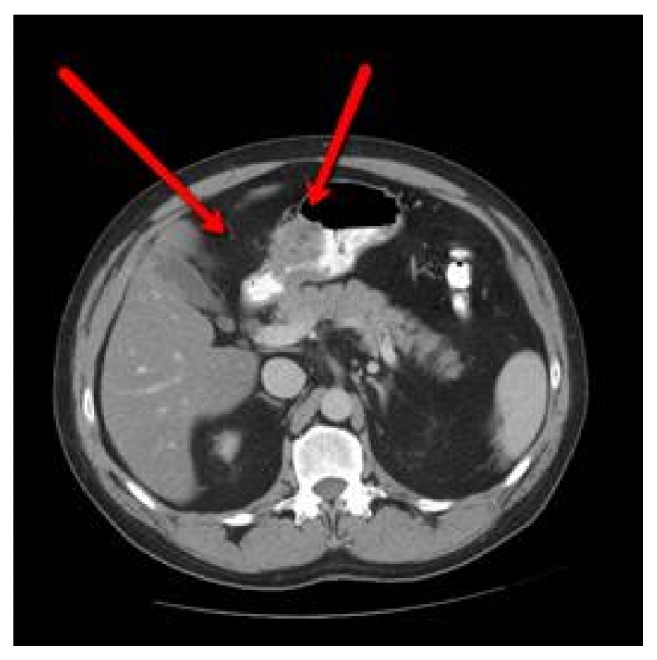
CT scan with IV contrast. Perigastric inflammatory changes are minimal (left arrow). Focal heterogeneous gastric thickening, consistent with residual inflammatory changes (right arrow).

He felt well for four months and then developed recurrent mild, diffuse, sharp, steady abdominal pain. He denied weight loss, nausea, vomiting, or bowel complaints. Physical examination showed normal vital signs, afebrile. His abdomen was soft, nontender, and otherwise unremarkable. Basic laboratory studies, serum amylase, and lipase were normal. Repeat upper GI endoscopy showed a firm submucosal mass with intact overlying mucosa and a central umbilication. Biopsy of the lesion revealed normal gastric mucosa (Figure 1). Abdominal CT with IV contrast (Figure 2) revealed minimal residual perigastric inflammatory changes (left arrow) and focal, heterogeneous gastric thickening, consistent with residual inflammatory changes (right arrow). Endoscopic ultrasound demonstrated an oval, intramural lesion 3.3 cm by 1.3 cm with irregular borders, which was aspirated by fine needle aspiration (FNA).

Findings were nondiagnostic, but inconsistent with leiomyoma or leiomyosarcoma.

**Figure 3:**
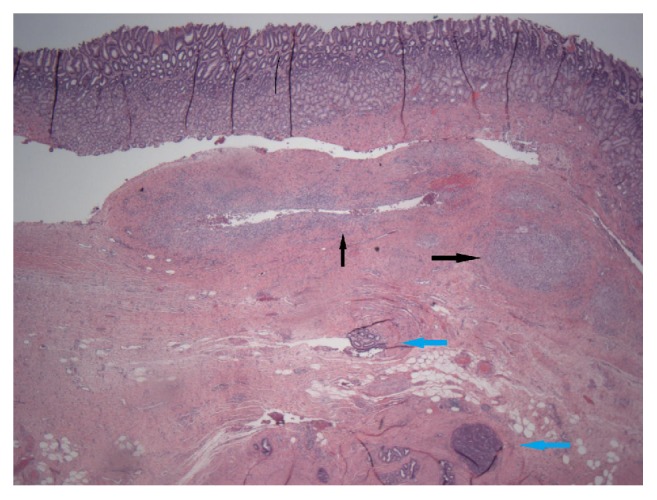
Gastric mucosa with underlying submucosa showing a small area of granulomatous inflammation (black arrows) and muscularis propria containing pancreatic ducts and acini (blue arrows) (H&E, 20x).

Open gastric antrectomy with a Billroth I technique was performed for a preoperative diagnosis of gastric adenocarcinoma. Histology of the resected specimen revealed ectopic pancreatic tissue, including excretory ducts, acini, and islet cells within the gastric muscularis layer (Figure 3). Evidence of chronic pancreatitis was present, including fibrosis and dilated ducts containing proteinaceous material. Also noted was an abscess believed to be related to focal acute pancreatitis in the ectopic tissue. At discharge, pain had resolved. He remained asymptomatic at 1-year follow-up.

## 3. Discussion

Heterotopic pancreas, also known as ectopic, accessory, or aberrant pancreas, or pancreatic rest, is defined as pancreatic tissue outside the normal pancreatic parenchyma in an aberrant location with a vascular and nerve supply separate from the pancreas itself [1]. It is thought to arise in fetal development during separation of the pancreatic tissue buds during foregut rotation and fusion of the dorsal and ventral pancreatic buds [1, 2]. At this stage, pancreatic buds are in contiguity with the distal stomach and proximal duodenum. The consequence may be dissemination of pancreatic tissue to ectopic sites. However, the finding of pancreatic tissue at distant sites such as the mediastinum and lungs has led others to explore alternative theories such as pancreatic tissue metaplasia [1].

Among patients with symptomatic gastric heterotopic pancreas, the most commonly reported symptom is mid-abdominal pain, occurring in as many as three-quarters of cases [2, 8]. Nausea, heartburn, dyspepsia, diarrhea, weight loss, hematemesis, and melena are described infrequently [1, 9, 10].

Reported complications of gastric ectopic pancreas include acute or chronic pancreatitis [4, 5, 7], pancreatic necrosis [5], pseudocyst formation [4, 7], gastric outlet obstruction [9], perforation [6], and, rarely, pancreatic carcinoma [11, 12].

Delayed or missed diagnosis is frequent [2, 10]. Factors implicated include (1) diverse, nonspecific complaints ranging from mild to severe, which may be indistinguishable from more common infectious, inflammatory, or neoplastic diseases [8, 9]; (2) rarity of clinical expression limits diagnostic suspicion of this entity [1, 4, 6]; (3) location in the submucosa or muscularis, typically with normal overlying mucosa, hampers diagnosis by standard endoscopy or imaging [4, 7]; (4) macroscopic appearance of heterotopic pancreas is pleomorphic [1], and it can be confused with a myriad of submucosal mass lesions; (5) classic endoscopic appearance of an antral submucosal bulge, which may contain a “crater-like” central umbilication corresponding to a draining duct, is absent in 50-80% of cases [1, 8]; (6) inflamed ectopic pancreatic tissue rarely produces sufficient enzymes to elevate serum amylase or lipase above normal [5].

Upper GI endoscopy and CT scanning are the most useful techniques to evaluate the majority of patients. The diagnosis of a submucosal mass lesion in the upper gastrointestinal tract with normal overlying mucosa on endoscopy can be divided into benign and malignant etiologies. Benign lesions include leiomyoma, lipoma, varices, neural tumors (i.e., schwannoma, neuroma, or neurofibroma), granular cell tumor, inflammatory fibroid polyp, duplication cyst, lymphangioma, Brunner's gland hyperplasia, benign GIST, and heterotopic pancreas. Malignant lesions that should be considered are malignant GIST, carcinoid tumor, lymphoma, glomus tumor, and gastric or metastatic carcinoma (especially melanoma, breast, lung, kidney, or ovarian cancer) [8, 9].

Endoscopic ultrasound (EUS) is the most accurate study to differentiate submucosal lesions not diagnosed by conventional endoscopy. Typical findings in heterotopic pancreas are a hypoechoic lesion with indistinct borders [8]. Uncommonly, EUS reveals anechoic duct-like structures [9]. An advantage of EUS is the ability to obtain guided biopsies for histologic evaluation. In our patient, EUS was nondiagnostic. When FNA is inconclusive, techniques other than the standard tissue biopsy may increase diagnostic yield. The success of routine EUS-guided biopsy using either standard or the more invasive jumbo forceps, the diagnostic yield in heterotopic pancreas, has been as low as 40% [1, 13]. The technique of endoscopic submucosal resection (ESMR) was found to improve diagnostic yield to 89% in a study by Hunt and colleagues [14].

Heterotopic pancreas may contain any of the components of normal pancreatic tissue, which consists of acini, ducts, and islets of Langerhans. In the case series of 32 patients reported by Ormarsson and colleagues, all cases contained ducts, 97% contained acini, and 41% contained islets [1]. Heterotopic pancreatic tissue can be divided into three histologic groups according to Heinrich's classification: (1) Type I is tissue with acini, excretory ducts, and islets of Langerhans; (2) Type II tissue contains acini and ducts, but no islets; (3) Type III tissue consists of ducts only. Type II is the most common of these types overall. However, Type I was the most common in 17 reported cases of HP-associated acute pancreatitis reviewed by Hamabe and colleagues [5]. Clinical pancreatitis occurring in heterotopic tissue is very rare. Type I tissue contains all components of normal pancreatic tissue; therefore it may be more susceptible to the acute pancreatitis seen in the anatomically normal pancreas [1, 5].

## 4. Conclusion

The diagnosis of symptomatic gastric heterotopic pancreatitis is challenging. This disorder, rarely detected ante-mortem, should be considered in selected patients with submucosal gastric mass lesions. We describe a patient with histologic confirmation of gastric ectopic pancreatic tissue including excretory ducts, acini, and islet cells within the gastric muscularis layer. Histologic acute and chronic pancreatitis with fibrosis, dilated ducts containing proteinaceous material and abscess formation, as in our patient, is exceptional. This disorder commonly presents with nonspecific symptoms, mimicking more common diseases. Nondiagnostic imaging and endoscopic findings are common, contributing to delay in diagnosis.
